# Exercise-Induced Regulation of Spexin: Implications for Metabolic Health: A Systematic Review and Meta-Analysis

**DOI:** 10.3390/medicina62010107

**Published:** 2026-01-03

**Authors:** İsa Aydemir, Yavuz Yasul, Taner Akbulut, Vedat Cinar, Gian Mario Migliaccio

**Affiliations:** 1 Department of Sport Management, Faculty of Sport Sciences, Hakkari University, Hakkari 30000, Turkey; isaaydemir@hakkari.edu.tr; 2 Bafra Vocational School, Ondokuz Mayıs University, Samsun 55400, Turkey; yavuz.yasul@omu.edu.tr; 3 Department of Coaching Education, Faculty of Sport Sciences, Firat University, Elazig 23119, Turkey; 4 Department of Physical Education and Sports Teaching, Faculty of Sport Sciences, Firat University, Elazig 23119, Turkey; vcinar@firat.edu.tr; 5 Department of Human Sciences and Promotion of the Quality of Life, San Raffaele Rome Open University, 00100 Rome, Italy

**Keywords:** exercise, metabolic health, obesity, spexin, diabetes

## Abstract

*Background/Objectives*: Spexin (SPX) is a bioactive peptide involved in the regulation of appetite, lipid metabolism, and glucose homeostasis. This systematic review and meta-analysis aimed to evaluate exercise-induced changes in SPX levels and their implications for metabolic health. *Methods*: This systematic review and meta-analysis aimed to synthesize evidence retrieved from PubMed, Web of Science, and Scopus databases, without restrictions on publication year, with the final literature search completed on 10 September 2024 and conducted in line with PRISMA 2020 reporting standards. The search strategy employed the keywords exercise, metabolic health, obesity, spexin and diabetes yielding 42 eligible records. Eligible studies included human or experimental animal populations exposed to acute or chronic exercise interventions. Exercise interventions included aerobic, resistance, combined, and high-intensity interval training protocols, with exercise intensity reported using heterogeneous metrics. The primary focus was on circulating SPX, alongside the assessment of related metabolic and endocrine parameters. Six studies satisfied the eligibility criteria and were included in the review. *Results*: The included studies were conducted in overweight or obese sedentary populations. Plasma SPX levels remained unchanged following acute (<3 weeks) aerobic exercise, whereas increased SPX levels were reported after chronic (≥3 weeks) exercise interventions. Elevated SPX concentrations were observed across different exercise modalities, including aerobic exercise, combined aerobic–resistance training, treadmill running, swimming, and HIIT. In addition to SPX, the included studies reported changes in metabolic and endocrine markers, including lipid-related variables, insulin-associated indices, adipokines, hormones, and selected metabolic proteins. *Conclusions*: This systematic review and meta-analysis indicate that exercise-related increases SPX are reported alongside changes in adiposity and metabolic–endocrine markers.

## 1. Introduction

Spexin (SPX) is a 14–amino acid peptide hormone first identified in 2007 [1]. It is recognized as a neuropeptide involved in multiple physiological processes, particularly energy homeostasis and metabolic regulation. Accumulating evidence indicates that SPX is closely associated with insulin and glucose metabolism, suggesting a complex role in the regulation of appetite, body weight, and overall metabolic balance [2,3,4]. Experimental studies have demonstrated that increased SPX levels suppress excessive food intake, reduce body weight, and enhance energy expenditure and lipolysis, underscoring its significance within the endocrine system and its relevance to metabolic disorders such as obesity and diabetes [5,6].

Beyond its established endocrine functions, SPX has recently attracted attention as a potential mediator of exercise-related metabolic adaptations. Several physiological effects attributed to SPX, such as improved glucose handling, enhanced insulin sensitivity, and increased energy expenditure, parallel the well-documented benefits of regular physical activity, suggesting a functional intersection between SPX signaling and exercise-induced metabolic regulation [7,8]. Consistent with these observations, exercise-induced secretion of SPX contributes to metabolic homeostasis, thereby supporting its classification as an exercise-responsive peptide implicated in metabolic adaptation [9].

Emerging evidence highlights the involvement of SPX signaling in the metabolic adaptations of skeletal muscle in response to exercise. Leciejewska et al. have reported, primarily in experimental settings, that elevated SPX levels stimulate myoblast proliferation and differentiation via the activation of GalR2 and GalR3 receptors, which are upregulated following exercise-induced stimuli [10]. Physical activity modifies sphingolipid metabolism, thereby enhancing glucose uptake in skeletal muscle, a process that may intersect with SPX-dependent signaling pathways [11]. SPX appears to orchestrate exercise-induced metabolic adaptations through two primary mechanisms. SPX activates GalR2 and GalR3 receptors in skeletal muscle, thereby promoting key myogenic processes that support muscle regeneration and adaptation. It also influences lipid-derived signaling cascades, notably through ceramide regulation and sphingomyelin hydrolysis, contributing to improved insulin responsiveness and enhanced glucose utilization. Beyond these local effects, SPX exerts systemic actions that further reinforce metabolic homeostasis. These include elevated energy expenditure and reduced appetite, both of which are critical factors in maintaining long-term energy balance [12,13,14]. These observations position SPX as a mediator of exercise-associated metabolic effects, with potential relevance in populations with compromised metabolic profiles. Indeed, resistance training has been shown to increase circulating SPX levels in individuals with obesity and type 2 diabetes, highlighting its relevance in clinical contexts [15,16].

Although the studies have examined exercise-induced changes in SPX levels, the findings remain inconclusive due to variability in study designs and population characteristics. Variations in exercise type, intensity, duration, and participant characteristics, reflecting the distinct metabolic demands of different exercise modalities, hinder direct comparisons of exercise-associated changes in SPX levels. This systematic review and meta-analysis aim to consolidate and quantitatively evaluate the current evidence on the impact of exercise on SPX levels, with a particular emphasis on its potential role in metabolic health, especially in relation to obesity.

## 2. Materials and Methods

### 2.1. Study Design

This systematic review was conducted in accordance with the Preferred Reporting Items for Systematic Reviews and Meta-Analyses (PRISMA) guidelines [17]. The study protocol was registered in the PROSPERO database (Registration No: 608955). The literature search was performed in the PubMed, Web of Science, and Scopus databases using predefined keywords related to exercise, SPX, metabolic health, obesity, and diabetes, combined with Boolean operators (AND/OR), covering studies published up to 10 September 2024.

### 2.2. Study Selection Process

**Figure 1:**
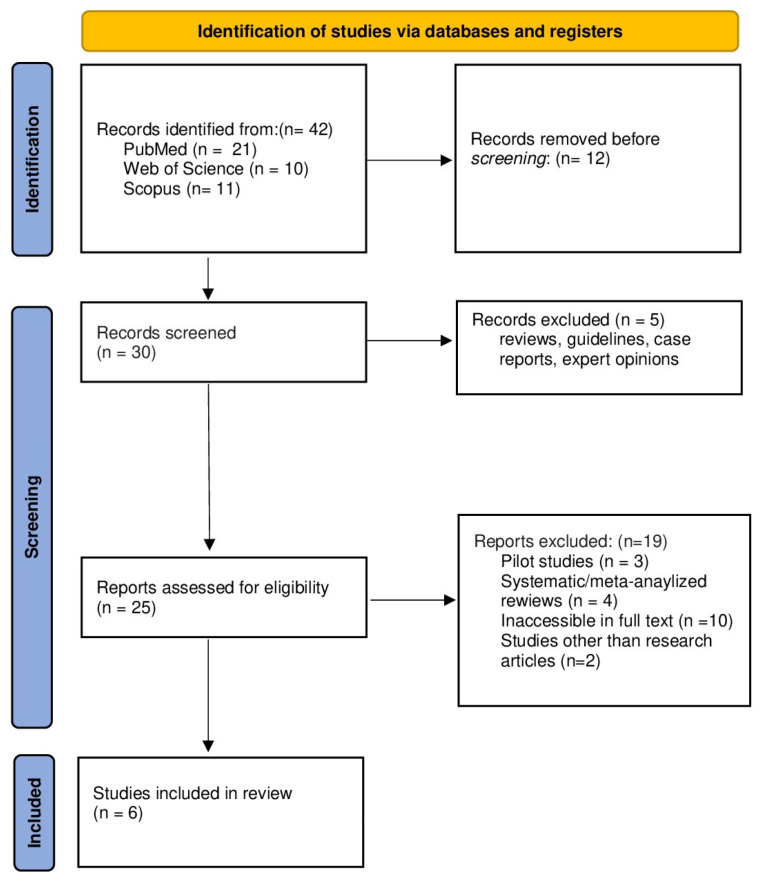
PRISMA 2020 flow diagram, which outlines the study selection process including identification, screening, eligibility, and inclusion, is illustrated.

Study selection was conducted using predefined inclusion and exclusion criteria targeting studies that examined exercise interventions alongside plasma SPX responses in the context of metabolic health, obesity, and diabetes. Eligibility criteria were based on population (human participants or rat models), intervention (structured exercise programs), and outcomes (plasma SPX levels). Original research meeting these criteria was included. Studies were excluded if they comprised reviews, meta-analyses, conference abstracts, case reports, expert opinions, pilot studies, non-peer-reviewed publications, or lacked accessible full texts. Through the database search process, 42 records were identified and subsequently subjected to title and abstract screening, which was independently performed by IA and YY. This initial screening resulted in six studies advancing to eligibility assessment, while the remaining records were excluded due to duplication, ineligible study designs, insufficient outcome reporting, or lack of relevance to the predefined criteria. Full-text assessments were independently conducted by two reviewers, and studies that fulfilled all eligibility criteria were included in the final synthesis. Discrepancies during the selection process were addressed through discussion. When consensus was not achieved, VC, TA, and GMM provided adjudication to determine the final inclusion decision. Ultimately, six studies satisfied the inclusion criteria. The study selection process is summarized in the PRISMA flow diagram (Figure 1).

### 2.3. Data Extraction

Data extraction was performed independently by researchers (IA and YY) using a standardized data extraction form. The following information was collected from each eligible study: author(s) and year of publication, study design, participant characteristics (species, sample size, gender, age, BMI category, and health status), exercise intervention characteristics (exercise type, modality, duration, and intensity/load), and primary outcomes related to plasma SPX levels. Additional extracted outcomes included changes in body composition, lipid profile, glycemic markers, hormones, adipokines, proteins, and enzymes where reported.

### 2.4. Quality Assessment

**Figure 2:**
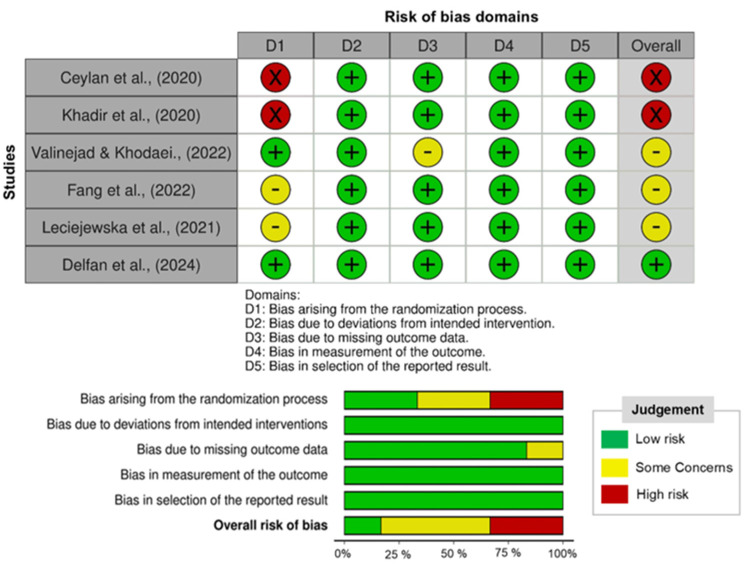
Risk of bias of the trials included in the present study is illustrated [10,15,16,19,20,21].

The methodological quality of studies was assessed using the Cochrane Risk of Bias tool [18], evaluating domains such as random sequence generation, allocation concealment, blinding, incomplete outcome data, and selective reporting. Risk of bias was categorized as low, moderate, or high according to the predefined assessment domains (Figure 2). Methodological variability, such as differences in sample size and exercise protocols, was acknowledged as a potential source of heterogeneity in interpreting the findings. While risk of bias ratings was not employed as exclusion criteria, they were taken into account during result interpretation.

### 2.5. Data Analysis

Meta-analysis was performed using Jamovi (version 2.7.14) with the meta-analysis module for continuous outcomes. Baseline-to-post-intervention changes were synthesized using raw mean differences (RMDs) and corresponding 95% confidence intervals (CIs), derived by computing the difference between post- and pre-intervention values. Estimates were pooled under a fixed-effects model, with study weights derived from inverse-variance estimation (model-based fixed-effects weights). Heterogeneity was assessed via Cochran’s Q test, as operationalized in Jamovi, and quantified using the I^2^ statistic to indicate the proportion of variance attributable to between-study differences. Substantial heterogeneity was observed (Q(3) = 15.86, *p* = 0.001; I^2^ = 81.1%), with between-study variance estimated at τ^2^ = 0. Model robustness was evaluated using standardized residuals and influence diagnostics, while potential publication bias was assessed through both regression- and rank-based approaches. Statistical significance was defined at a two-sided α level of 0.05 [19,20].

## 3. Results

### 3.1. Study Characteristics

Six studies were identified and critically evaluated [10,15,16,21,22,23]. The study designs included intervention trials with measurements obtained at two distinct time points, randomized intervention-controlled trials, and randomized experimental controlled designs. Intervention trials involved sedentary adult male participants (*n* = 20) classified as overweight or obese, while intervention-controlled studies included mixed sex adult populations (*n* = 185) comprising individuals with obesity and obesity with type 2 diabetes mellitus. Randomized, double blind intervention-controlled trials enrolled male participants (*n* = 36) who were overweight or obese, and one randomized, double-blind, placebo-controlled study included male participants with obesity (*n* = 44). Experimental studies involved obese rat models and applied randomized, experimental controlled designs (Table 1).

### 3.2. Exercise Interventions

Exercise protocols exhibited substantial heterogeneity with respect to modality, duration, and intensity. Both acute and chronic interventions were employed, with chronic exercise programs ranging from 3 to 12 weeks in duration. Aerobic exercise constituted the most frequently implemented modality, followed by combined aerobic–resistance training and HIIT. In animal-based studies, swimming and treadmill running served as the primary exercise models. Exercise intensity in human studies was predominantly prescribed using heart rate–based indices or VO_2_max-derived workload equivalents. Aerobic exercise intensities ranged from low-to-moderate (55–59% of maximum heart rate) to moderate-to-vigorous levels (65–80% of maximum heart rate). Combined aerobic and resistance exercise protocols typically involved aerobic training performed at 60–70% of maximum heart rate alongside resistance training at intensities corresponding to 90–95% of maximal effort. The HIIT protocol followed a progressive loading strategy, with exercise intensity increasing from approximately 75% of maximum heart rate during the initial weeks to 95% in the final phase of the intervention. In animal models, exercise load was standardized as either swimming for 60 s per day or treadmill running at a constant speed of 12 m/min with a 5% incline.

### 3.3. Exercise-Induced Spexin Outcomes

Plasma SPX concentration was assessed in all included studies. Plasma SPX levels did not change following an acute aerobic exercise session performed at 55–59% heart rate. Plasma SPX levels were higher following a 12-week aerobic exercise intervention conducted at 65–80% heart rate and after a 6-week combined aerobic and resistance exercise program involving aerobic exercise at 60–70% heart rate and resistance exercise at 90–95% heart rate. Plasma SPX levels were also higher following a 3-week swimming protocol performed for 60 s per day, a 4-week treadmill running intervention at a speed of 12 m/min with a 5% incline, and a 12-week HIIT program applying progressively increasing exercise intensity from 75% to 95% heart rate.

### 3.4. Metabolic and Endocrine Outcomes

Exercise interventions were associated with coordinated metabolic and endocrine adaptations. Endocrine-related markers, including asprosin, insulin, leptin, and lipocalin-2, decreased, whereas ghrelin levels increased. Metabolic and body composition outcomes, including total cholesterol (TC), triglyceride (TG), body mass, body mass index (BMI), visceral fat, and homeostasis model assessment of insulin resistance (HOMA-IR), decreased, while high-density lipoprotein cholesterol (HDL-C) and lean body mass (LBM) increased. Markers related to metabolic regulation, including glucose transporter type 4 (GLUT4), sirtuin 1 (SIRT1), and peroxisome proliferator-activated receptor gamma coactivator 1-alpha (PGC-1α), increased.

### 3.5. Meta-Analysis of Changes in SPX Levels

**Figure 3:**
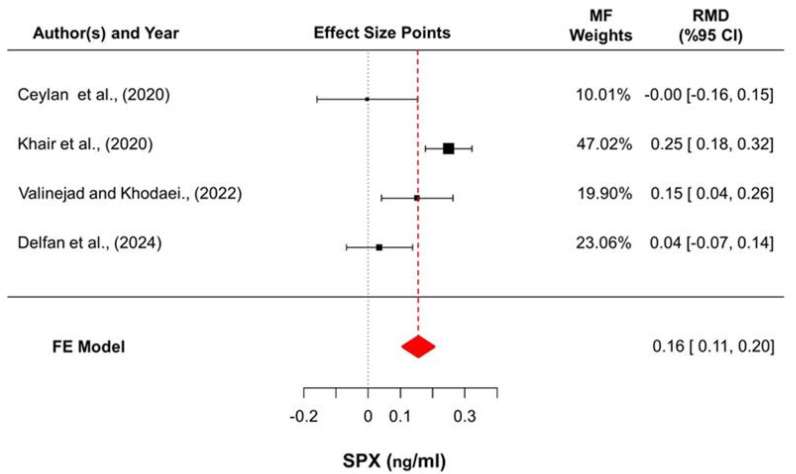
Forest plot summarizing the pooled changes in SPX concentrations. Effect sizes are presented as raw mean differences (RMDs) with 95% confidence intervals, calculated as post-intervention minus pre-intervention values. Square sizes reflect inverse-variance-based fixed-effects weights, and horizontal lines denote confidence intervals. The diamond represents the overall pooled estimate derived from the fixed-effects (FE) model [16,19,20,21].

The meta-analysis yielded a statistically significant pooled increase in SPX levels, with a raw mean difference of 0.156 ng/mL (95% CI: 0.106–0.205; z = 6.17; *p* < 0.001) based on a fixed-effects model. Reported estimates varied from −0.003 to 0.250 ng/mL, and positive changes were observed in three of the four studies (75%). Substantial heterogeneity was detected (Q (3) = 15.86; *p* = 0.001; I^2^ = 81.1%), although the estimated between-study variance was negligible (τ^2^ = 0). Evaluation of standardized residuals indicated that [16,23] may represent potential statistical outliers; however, influence diagnostics confirmed that no individual study exerted a disproportionate impact on the pooled effect estimate (Figure 3).

## 4. Discussion

The release of metabolic regulatory peptides, including irisin, fibroblast growth factor 21 (FGF21), and SPX is triggered by exercise and plays a role in the regulation of energy balance and metabolic processes within the body. The secretion of irisin in skeletal muscle in response to exercise-induced stimuli contributes to the management of body weight through increased energy expenditure. Irisin, in response to exercise, affects the levels of PGC-1α in skeletal muscle, thereby maintaining glucose homeostasis and promoting the browning of white adipose tissue. Furthermore, irisin injections are regarded as a viable alternative to exercise for obese patients [24,25]. The exercise-induced increase in FGF21 secretion affects energy homeostasis and lipid metabolism by increasing insulin sensitivity in the liver. This relationship between FGF21 and metabolic parameters is particularly linked to improvements in insulin sensitivity and hepatic lipid metabolism [26]. Whereas irisin and FGF21 are primarily recognized for their roles as exercise-induced peptides involved in metabolic regulation, SPX demonstrates a broader functional spectrum as a pleiotropic neuropeptide, exerting significant endocrine and paracrine influences on appetite control, glucose homeostasis, and lipid metabolism through GalR2- and GalR3-mediated pathways [27,28]. Although each of these bioactive molecules responds to the metabolic stress associated with physical activity, SPX uniquely integrates both central and peripheral metabolic signals. This integrative property distinguishes it from classical myokines and hepatokines, positioning SPX as a discrete regulatory entity within the exercise–metabolism interface. Accumulating evidence indicates that molecules such as irisin and FGF21 serve as key transducers converting mechanical and metabolic stimuli into coordinated systemic adaptations. Exercise-responsive signaling frameworks position SPX as a mediator operating through functionally distinct yet complementary mechanisms involved in energy homeostasis [10,15,29].

SPX’s regulatory roles in energy balance, as well as glucose and lipid metabolism, facilitate the development of innovative therapeutic approaches for treating chronic conditions such as obesity, diabetes, and cardiovascular diseases [30]. SPX is closely associated with insulin and glucose. Both insulin and glucose can be stimulated to modulate SPX, while SPX, in turn, can regulate insulin. This interplay may represent a potential mechanism linking insulin to the regulation of blood glucose levels [31]. Research indicates that exercise can alter SPX levels, significantly affecting metabolic health. While low SPX levels are observed in metabolic disorders such as obesity and type 2 diabetes (T2DM), exercise can raise SPX levels, improving insulin sensitivity and glucose tolerance [32,33]. Regular aerobic exercise at moderate to high intensity has been associated with increases in circulating SPX levels and improvements in insulin sensitivity; however, the available evidence is largely based on studies with small sample sizes and heterogeneous exercise protocols. The present meta-analysis identified a significant pooled increase in SPX levels following exercise interventions, while also demonstrating substantial between-study heterogeneity, suggesting that exercise-induced SPX responses are not uniform across study designs or training modalities. Consequently, definitive conclusions regarding dose–response relationships between exercise intensity, duration, and SPX regulation cannot yet be drawn, highlighting the need for more rigorous and quantitative synthesis of existing data [34,35]. Short-term exercise training, on the other hand, improves glucose metabolism by providing temporary increases in SPX levels. Furthermore, exercise stimulates adipo-myokines (hormones secreted by adipose and muscle tissue), supporting energy homeostasis and regulation of glucose metabolism. In addition, exercise increases SPX levels in older individuals and protects them against age-related metabolic diseases [15].

Studies involving obese individuals have demonstrated reduced levels of SPX, with the hypothesis that these levels could be increased through exercise interventions. The findings indicated that exercise-induced elevations in SPX levels were evident among obese participants. Similarly, physical activity was shown to increase SPX levels in individuals with T2DM, contributing to improved glucose metabolism. Furthermore, a 12-week physical activity program performed at 65–80% of maximum heart rate led to elevated SPX levels in previously sedentary adult men and women, highlighting the relationship between exercise and SPX regulation [33,34,35,36]. Ceylan et al. performed two acute exercise interventions at 55–59% heart rate in sedentary men and reported no change in SPX levels [21]. Consistent with these observations, the meta-analytic findings indicate that positive SPX responses were predominantly observed in studies employing chronic exercise interventions, whereas acute protocols contributed limited or null effects to the pooled estimate. When the evidence is considered collectively, a clear distinction emerges between acute (<3 weeks) and chronic (≥3 weeks) exercise interventions. Acute exercise typically does not produce consistent alterations in circulating SPX levels, particularly when performed at lower intensity or for short durations in contrast, chronic exercise training conducted over several weeks, especially at moderate to high intensity, is more frequently associated with increased SPX concentrations. This contrast indicates that SPX regulation is more closely related to sustained metabolic stress and cumulative physiological adaptations than to transient exercise stimuli [23,36]. Accordingly, divergent findings across studies appear to reflect differences in intervention duration rather than methodological inconsistency. This response pattern is consistent with that observed for other exercise responsive peptides, which generally require repeated metabolic challenges to elicit stable endocrine adaptations.

Alterations in circulating SPX levels have primarily been reported in studies utilizing moderate- to high-intensity, long-duration exercise interventions. Aerobic training protocols conducted at approximately 60–70% of maximum heart rate, as well as resistance training performed at 90–95% intensity over periods ranging from 6 to 12 weeks, have been shown to significantly increase SPX concentrations. On the other hand, low-intensity exercise routines and isolated acute bouts of physical activity typically do not lead to notable changes in SPX levels, particularly among sedentary or metabolically healthy individuals. In contrast, evidence from overweight and obese populations indicates that a sufficient exercise stimulus, achieved through higher-intensity aerobic and resistance-based programs, is required to elicit significant increases in SPX responses [10,15,22,37]. These outcomes indicate that chronic exercise interventions provide a more reliable basis for evaluating SPX responsiveness than acute exercise models. However, this conclusion should be interpreted with caution due to substantial variability in exercise intensity, participant characteristics, and SPX measurement methodologies across existing studies [23].

## 5. Limitations

This systematic review and meta-analysis is subject to several methodological limitations that warrant consideration when interpreting the findings. The evidence base is defined by the number of eligible studies, particularly in human interventions, which may influence the precision of effect estimates. Variation in exercise modality, intervention duration, and training intensity introduces heterogeneity that should be considered when comparing outcomes. The included studies primarily focused on overweight or obese populations and more frequently involved male participants or animal models, which may constrain generalizability. In addition, SPX measurements were performed under varying experimental conditions, as assay procedures and sampling protocols were not fully standardized across studies. Certain lifestyle related factors, such as dietary intake and habitual physical activity, were not consistently reported and therefore could not be systematically evaluated. Despite these constraints, the availability of sufficient data to permit quantitative synthesis represents a key strength of the present work, allowing the first meta-analytic evaluation of exercise associated SPX responses and providing a structured framework to inform and guide future research.

## 6. Conclusions

Long-term exercise interventions were more consistently associated with increases in circulating SPX levels than isolated acute exercise bouts. This conclusion is supported by both qualitative synthesis and the meta-analysis, which demonstrated a significant pooled increase in SPX levels following exercise interventions (raw mean difference: 0.156 ng/mL, 95% CI: 0.106–0.205; *p* < 0.001), despite substantial heterogeneity across studies. The influence analysis showed that no single study had a disproportionate effect on the overall effect estimate. On the other hand, SPX responses to acute exercise were inconsistent, with unchanged SPX levels reported following moderate-intensity acute aerobic exercise performed at 55–59% of maximum heart rate. Evidence on high-intensity acute exercise remains limited, thereby restricting definitive conclusions about intensity-dependent SPX responses. Thus, differences between chronic and acute exercise responses should be regarded as indicative patterns rather than definitive mechanistic outcomes.

Exercise interventions implemented in individuals with overweight, obesity, or type 2 diabetes were more consistently associated with elevated SPX levels, suggesting that baseline metabolic status may influence SPX responsiveness. Reported exercise intensities varied substantially across studies, ranging from approximately 55–59% to 95% of maximum heart rate. This variation limits comparisons of SPX results across studies and points to the need for clearer intensity definitions. In addition to, human studies have largely been limited to sedentary individuals, leaving SPX responses in physically active populations and trained athletes poorly understood. While aerobic exercise and high-intensity interval training have commonly been associated with increased SPX levels, evidence on resistance-based exercise remains limited, particularly in relation to progressive loading and total training volume. Understanding this gap may help clarify whether SPX can be used as a marker of training load or physical adaptation in response to resistance exercise.

### Future Directions

Future studies should standardize exercise intensity and reporting methods to enhance comparability across SPX-related research. Investigations involving physically active individuals and trained athletes are also needed to clarify SPX responses beyond sedentary populations. Moreover, well-controlled trials on resistance exercise with systematic adjustments in load, volume, and progression may help determine whether SPX functions as a marker of training adaptation or metabolic stress. Finally, mechanistic studies examining SPX signaling pathways and receptor activity could provide deeper insight into its role in exercise-induced metabolic regulation.

## Figures and Tables

**Table 1:** Study characteristics and outcomes are presented.

Authors, Year	Research Design	Gender	Participant	Exercise Type and Duration	Exercise Load Vo_2max_ (mL/kg/min.)	Plasma Spexin Level	Others Results		
							Lipids	Hormones	Adipokines Proteins Enzyme
Leciejewska et al., [10]	Randomized, experimental-controlled design	Rats	Obese	Treadmill Running Exercise 4-week	Rats ran at a speed of 12 m/min (5% slope)	Rised ↑			GalR2 ↑ GalR3 ↔
Fang et al., [15]	Randomized, experimental-controlled design	Rats	Obese	Aerobic Exercise 3-week	Swimming, 60 s per day	Rised ↑	Body Mass ↓ Visceral Fat ↓ TG ↓ TC ↓	Insulin ↓	GLUT4 ↑ SIRT1 ↑ PGC-1α ↑
Khadir et al., [16]	Two trials intervention-controlled design	Adult Female/Male (n = 185)	Obese Obese with T2DM	Aerobic Exercise 12-week	65–80% heart rate	Rised ↑	TC ↓ HDL-C ↑ HOMA-IR ↓		
Ceylan et al., [21]	Two trials, intervention design	Adult Male (n = 20)	Overweight Obese	Aerobic Exercise Acute	55–59% heart rate	unchanged ↔		Asprosin ↓ Insulin ↓	Lipocalin-2 ↓
Valinejad & Khodaei., [22]	Two trials, randomized, double-blind, intervention-controlled design	Male (n = 36)	Overweight Obese	Aerobic and Resistance Exercise 6-week	Aerobic training: 60–70% heart rate. Resistance training 90–95% heart rate.	Rised ↑	Body Mass ↓ BMI ↓ LBM ↑	Leptin ↓ Ghrelin ↑	
Delfan et al., [23]	Two trials, randomized, double-blind and placebo-controlled design	Male (n = 44)	Obese	HIIT 12-week	1–2 weeks: 75% heart rate. 3–4 weeks: 85% heart rate. 5–10 weeks: 90% heart rate. 11–12 weeks: 95% heart rate.	Rised ↑		Asprosin ↓ Irisin ↑	Lipocalin-2 ↓ Omentin-1 ↑

**Table 1**. Study characteristics and exercise-associated changes in plasma spexin and related metabolic outcomes are summarized. Abbreviations; HIIT: high-intensity interval training, TC: total cholesterol, HDL-C: high-density lipoprotein, HOMA-IR: Homeostasis model assessment of insulin resistance, BMI: Body mass index, LBM: Lean body mass, TG: Triglyceride, GLUT4: glucose transporters type 4, SIRT1: sirtuin 1, PGC-1α: peroxisome proliferator-activated receptor-γ co-activator-1α, GalR2: galanin receptor 2, GalR3: galanin receptor 3, Vo2max: maximal oxygen consumption, min: minute.

## Data Availability

No new data were created or analyzed in this study. Data sharing is not applicable to this article as all data were obtained from previously published and publicly available sources.
